# Higher illness burden is associated with reduced heart rate variability in bipolar disorder

**DOI:** 10.1192/j.eurpsy.2021.233

**Published:** 2021-08-13

**Authors:** A. Ortiz, I. Husain, P. Moorti, M. Alda, M. Sanches, B. Mulsant

**Affiliations:** 1 Department Of Psychiatry, University of Toronto, Toronto, Canada; 2 Mood Disorders, The Royal Ottawa, Ottawa, Canada; 3 Psychiatry, Dalhousie University, Halifax, Canada

**Keywords:** bipolar, heart rate variability, illness burden

## Abstract

**Introduction:**

Bipolar disorder (BD) is associated with premature death and ischemic heart disease is the main cause of excess mortality. The predictive power of heart rate variability (HRV) for mortality has been confirmed in patients with or without cardiovascular disease. While several studies have analyzed the association between HRV and BD, their results are incongruent; and none has analyzed the effect of the clinical factors characterizing illness burden on HRV.

**Objectives:**

To assess the association between HRV and the following factors characterizing illness burden: illness duration, number and type of previous episode(s), duration of the most severe depressive or hypomanic/manic episode, severity of episodes, co-morbid psychiatric disorders, family history of BD or suicide, and duration and polarity of current episode in participants experiencing one.

**Methods:**

We used a wearable device in 53 BD participants to assess the association between HRV using 4 measures (RMSSD, SDANN, SDNN and RR Triangular Index) and the abovementioned clinical factors characterizing illness burden. For each of the 4 HRV measures we ran 11 models, one for each burden of illness clinical factor as an independent variable.

**Results:**

Longer illness duration, higher number of depressive episodes, and family history of suicide were negatively correlated with HRV; in the 14 participants experiencing a depressive episode, the MADRS score was negatively correlated with HRV

**Conclusions:**

Our study analyzed the association between burden of illness and HRV in BD, while controlling for functional cardiovascular status, age, sex, BMI, education, and treatment. Our results showed that high illness burden is associated with reduced HRV.

**Disclosure:**

No significant relationships.

